# Modelling and simulation of multicomponent acetone-butanol-ethanol distillation process in a sieve tray column

**DOI:** 10.1016/j.heliyon.2021.e06641

**Published:** 2021-04-08

**Authors:** Lily Pudjiastuti, Tri Widjaja, Kornelius Kevin Iskandar, Fikran Sahid, Siti Nurkhamidah, Ali Altway, Atha Pahlevi Putra

**Affiliations:** a Industrial Chemical Engineering Department, Institut Teknologi Sepuluh Nopember, Sukolilo, Surabaya 60111, Indonesia; b Chemical Engineering Department, Institut Teknologi Sepuluh Nopember, Sukolilo, Surabaya 60111, Indonesia

**Keywords:** Acetone-butanol-ethanol, Biobutanol, Distillation, Multi-component, Modelling, Simulation

## Abstract

Renewable energy sources are prospective solutions for addressing future energy needs arising from the ever-increasing population and dwindling petroleum reserves. Biobutanol is one of the most efficient biofuels for use as a mixture with motor vehicle fuels. Biobutanol is produced from the acetone-butanol-ethanol (ABE) fermentation process and is separated into the pure components via multicomponent distillation. Mathematical modelling of the continuous multicomponent distillation of ABE was carried herein out using an equilibrium-based model with the modified Hang-Wanke method in MATLAB R2020a programming language and compared with the simulation results using Aspen Plus V9. The variables of this study were the feed stage, number of trays, reflux ratio to butanol purity, butanol recovery, and energy load of the reboiler and condenser. Based on the simulation results, the operating conditions in columns 1 and 2 were recommended based on the butanol purity, recovery, and reboiler load; the recommended operating conditions for column 1 are as follows—feed stage: 4, reflux ratio: 4, number of trays: 20 trays, with a column efficiency of 55.43%. The recommended operating conditions for column 2 are as follows—feed stage: 2, reflux ratio: 0.4, number of trays: up to 10, with a column efficiency of 54.94%.

## 1 Introduction

Indonesia has a high energy demand due to the large population. According to data from the National Development Planning Agency (Bappenas) in 2013, in 2019, Indonesia's population reached 267 million and is expected to increase every year.

The increase in the energy demand is not proportional to the existing energy reserves. According to data from the Ministry of Energy and Mineral Resources (ESDM), the oil reserves in Indonesia in 2019 were only 3.2–3.3 billion barrels, and with the current production level of 803 thousand barrels per day, these reserves will only last for nine more years. Among the alternative energies being developed, biobutanol is a promising fuel. Compared to ethanol, its properties are more similar to those of gasoline. This is because butanol has a longer carbon chain than ethanol, resulting in a higher volatilization rate, combustion rate, and octane value. Butanol can also reduce the cost of using anti-corrosion materials when incorporated into certain systems and is safe for use in machines (Tracy et al., 2011). In addition, butanol can also be mixed with gasoline at any percentage, which makes butanol far superior to ethanol (Karimi et al., 2015).

Biobutanol is produced by biomass fermentation. When *Clostridium acetobutylicum* is fed with sugars produced from biomass, the microbes break down sugar into various types of alcohols, including acetone, ethanol, and butanol (National Energy Council of the Republic of Indonesia). The most commonly used biomass types are whey permeate (a by-product of the dairy industry that contains lactose), corn, wood hydrolysate, and other monomer sugars such as glucose (Qureshi and Blaschek, 2000).

Fermentation that produces biobutanol is commonly called acetone-butanol-ethanol (ABE) fermentation because, in addition to producing butanol, it also produces acetone and ethanol as the main products. In 1950, the use of this method was discontinued because of the switch to producing acetone and butanol from petroleum-based products because of the cheap price of petroleum in that period. Under the current conditions, with the depletion of petroleum reserves, the ABE fermentation method has regained attention because of its potential for producing butanol, which can be mixed with vehicle fuel (Ndaba et al., 2005).

During the last twenty years, experimental and computational studies have been carried out to increase the efficiency of the ABE fermentation process. Simulation is often used as the main tool in process engineering to scale up production and in research to provide accurate predictions of plant performance, and is also used in the development and optimisation of biobutanol production. The earliest research carried out in the downstream process of ABE fermentation using simulations was undertaken by Marlatt and Datta (1986) and Dadgar and Foutch (1988), who evaluated the economics of the biobutanol production process. This research was continued by many other researchers, for both the upstream (fermentation and biochemistry) and downstream (separation and purification) processes (Liu et al., 2009). In the upstream process, research on biochemistry and microorganisms (Linden et al., 1985; Ennis et al., 1986; Jones and Woods, 1986; Kharkwal et al., 2009; Ni and Sun, 2009) has been conducted to improve fermentation technology in order to produce ABE in greater concentrations and quantities. For the downstream process, various ABE separation methods such as gas stripping (Dadgar and Foutch, 1988; Park and Geng, 1992; Qureshi and Blaschek, 2001a, Qureshi and Blaschek, 2001b), column distillation (Marlatt and Datta, 1986; Qureshi and Blaschek, 2001a, Qureshi and Blaschek, 2001b; Sari et al., 2007; Luyben, 2008; Liu et al., 2009; Sari, 2011a, Sari, 2011b), extraction (Dadgar and Foutch, 1988), adsorption (Park and Geng, 1992), and pervaporation (Qureshi et al., 1992; Qureshi and Blaschek, 2001a, Qureshi and Blaschek, 2001b; Hickey and Slater, 1990) have been studied to find the best unit operation that can efficiently separate the ABE mixture into its pure components. Of the various separation methods, column distillation is still the main choice because the process is simpler and can produce higher purity products, although it requires greater energy. Patil et al. (2014) simulated the distillation of *iso*amyl acetate via reactive distillation (RD) using Aspen Plus® and MATLAB^(^™^)^. Lone (2015) simulated the pre-evaporation‒distillation of a MeOH and MTBE mixture with a distillation column using MATLAB ^(^™^)^. Haigh et al. (2018) simulated the separation of ABE and analysed its economic potential.

Most of the studies that have focused on the downstream process of ABE production highlighted economic analysis, the simulation process, and validating the simulation results. No detailed examination of the effect of the parameters of the distillation column on the product purity and energy use is documented. In fact, the distillation process is the most important operating unit for effective and efficient separation. Modelling and simulation of the ABE ternary continuous distillation process is required to assist with the design of separation processes to obtain the highest possible butanol quality in the industry under accurate and optimal operating conditions. Therefore, in this study, modelling and simulation of the distillation of the acetone-butanol-ethanol (ABE) ternary system in a sieve tray column is carried out by taking into account the effects of the feed stage parameters, reflux ratio, and the number of trays of the two distillation columns on the purity and recovery of butanol as the main product, and the energy load on the reboiler and condenser, respectively. Studies documenting simulation and modelling of ABE distillation are still very rare, and we compare simulations derived from the equilibrium-based model with the Hang-Wanke method using the MATLAB R2020a program with the simulation results using Aspen Plus V9 with the RadFrac distillation column. In using the Hang and Wanke method, we modified the convergence criteria and considered the tray efficiency. It is hoped that this research can provide a reference for the design and optimisation of processes in actual industries.

## 2 Materials and methods

Studies on butanol recovery via the simulation and modelling of ABE multicomponent continuous distillation on the sieve tray column have focused on the effect of the feed stage, reflux ratio, and operating pressure on the distillation column, among other factors.

**Figure 1:**
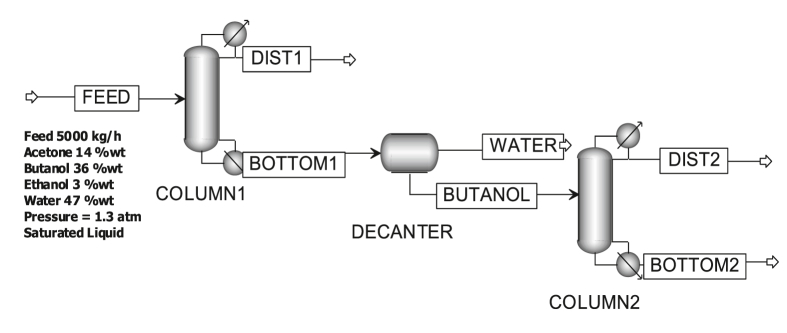
Acetone-butanol-ethanol multicomponent continuous distillation system.

The system reviewed herein is the distillation and decanter column system, as shown in Figure 1.

This system comprises two distillation columns and a decanter (Luyben, 2008; Doherty and Malone, 2001). The feed in this study is distillate from the broth distillation for separating organic acids from solvents, as reported by Marlatt and Datta (1986). The first column separates acetone-ethanol (AE) as a distillate and butanol-water (BW) as the bottom product. The decanters separate butanol-water (BW) mixtures, which form heterogeneous azeotropes (Luyben, 2008; Doherty and Malone, 2001; Marlatt and Datta, 1986). The second column functions to separate and purify butanol as the bottom product.

**Figure 2:**
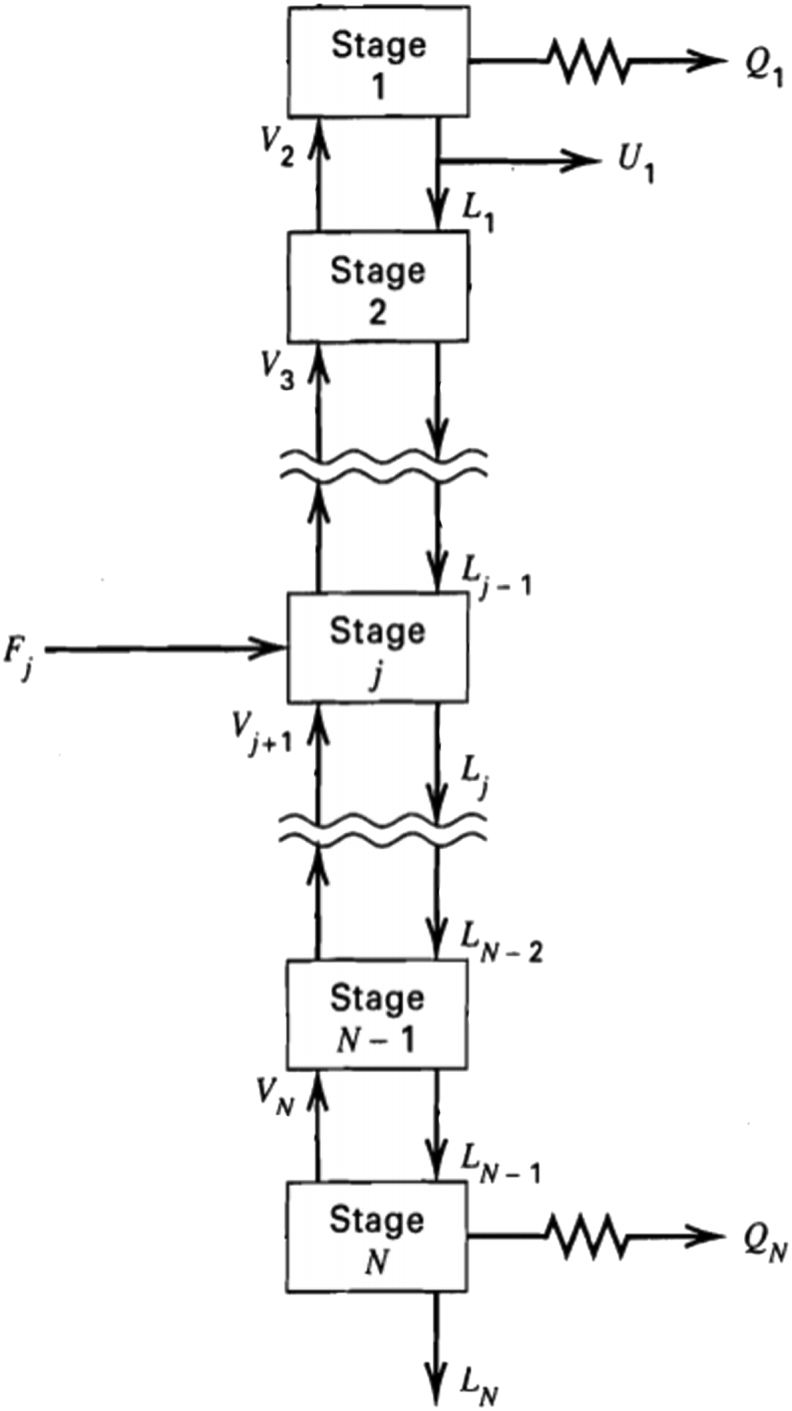
Acetone-butanol-ethanol multicomponent continuous distillation system.

The simulation was conducted using matrix laboratory software (MATLAB) to study the distillation process by developing a rigorous equilibrium-based method (Hang and Wanke method) for multicomponent and multistage distillations to describe the separation process. A schematic of the counter-current multicomponent and multistage distillation separation process carried out in this study is shown in Figure 2 with reference to the rigorous equilibrium-based method (Seader and dan Henley, 2006). Each stage is considered to have reached equilibrium. The model consists of MESH equations (Seader and dan Henley, 2006), which comprise the nonlinear algebraic equation system to be solved using the tearing variables (vapour rate and temperature) in each stage and using the bubble point method to predict the new temperature profile (Hang-Wanke method). Herein, the convergence criteria typically used in the Hang-Wanke method were modified.

The vapour‒liquid equilibrium data required in this simulation were estimated using the modified Roult Rule with the correction of nonideality in the liquid phase using the activity coefficient. The liquid phase activity coefficient for non-ideal mixed solutions must be predicted even when experimental phase equilibrium data are not available, and the assumption of regular solutions is invalid because of the polar components. Instead of basing this prediction on molecules, Wilson and Deal, then Derr and Deal, in 1960, introduced a method based on treating the solution as a functional group mixture. The larger the functional group, the more accurate the molecular representation, but the advantages of the group contribution method are reduced because a larger group is required. In practice, fifty functional groups are used to represent thousands of multicomponent liquid solution mixtures (Seader and dan Henley, 2006). Table 1 shows the UNIFAC group used in this study.

**Table 1:** Group UNIFAC specification.

Component (i)	Name	Main Group	Sec. Group	Rj	Qj
Acetone (1)	CH_3_	1	1	0.9011	0.848
	CH_3_CO	9	18	1.6724	1.488
Butanol (2)	CH_3_	1	1	0.9011	0.848
	CH_2_	1	2	0.6744	0.540
	OH	5	14	1.0000	1.200
Ethanol (3)	CH_3_	1	1	0.9011	0.848
	CH_2_	1	2	0.6744	0.540
	OH	5	14	1.0000	1.200
Water (4)	H_2_O	7	16	0.9200	1.400

(Polling, 2001).

The UNIFAC group contribution method (UNIQUAC Functional-group Activity Coefficients), first introduced by Fredenslund, Jones, and Prausnitz, has been continually developed, and has several advantages over other group contribution methods (Seader and dan Henley, 2006).

The UNIFAC method for predicting the liquid phase activity coefficient is based on the UNIQUAC equation. The UNIFAC parameters used in the modelling carried out in MATLAB are shown in Table 1, and the interaction parameters for each group are listed in Table 2.

**Table 2:** Interaction group parameter UNIFAC.

a (m, n)		n			
		1	5	7	9
m	1	0	986.5	1318	476.4
	5	156.4	0	353.5	84
	7	300	-229.1	0	-195.4
	9	26.76	164.5	472.5	0

(Polling, 2001).

The research variables evaluated herein include fixed, independent, and dependent variables. Table 3 lists the variables investigated in this study.

**Table 3:** Research variable.

Fixed	Independent	Dependent
Feed Rate = 5000 kg/h	**Column 1**	%Recovery Butanol
Feed Pressure = 1,3 atm	Reflux ratio = 3, 4, 5, 6	Kandungan Butanol
Acetone = 14%	Feed stage = 3, 4, 5, 6, 7, 8	Heat duty reboiler and condenser
n-Butanol = 36%	Number of trays = 20, 30, 40, 50, 60	
Ethanol = 3%	**Column 2**	
Water = 47%	Reflux ratio = 0.2, 0.4, 0.6, 0.8, 1	
q = 1	Feed stage = 2, 3, 4, 5, 6	
	Number of trays = 10, 20, 30, 40	

The data from the MATLAB R2020a program were compared with the simulation results using Aspen Plus V9 with the RadFrac distillation column model.

## 3 Result and discussion

Based on the simulation and modelling results, the effects of the feed stage, reflux ratio, and number of trays on the purity and recovery of *n*-butanol, as well as the energy load on the reboiler and condenser under atmospheric conditions, were evaluated.

The number of stages specified in this study is the number of theoretical stages, which is correlated with the number of actual stages by considering the overall column efficiency. The overall column efficiency correlates with the product of the relative volatility of the light key/heavy key component and the mean viscosity based on the feed composition in mole% at the mean column temperature. This approach has been shown to provide a reliable estimate of the overall column efficiency for a hydrocarbon system and can be used to estimate the efficiency for other systems. This method does not consider the design parameters of the plates, and includes only two physical property variables. Eduljee (1958) expressed O'Connell's correlation as Eq. (1) (Sinnot, 2005).

(1) *Eo* = 51–32.5 × log(μL × αa)

µL is the liquid viscosity in mPa-s and α is the relative volatility.

**Figure 3:**
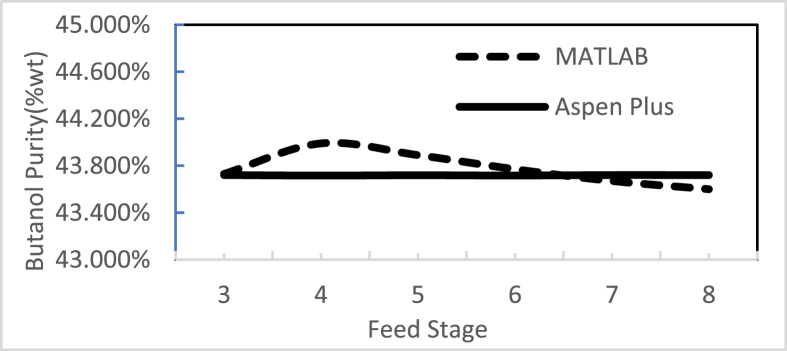
Effect of feed stage on the purity of butanol in column 1, R = 4, N = 20, D/F = 0.15.

**Figure 4:**
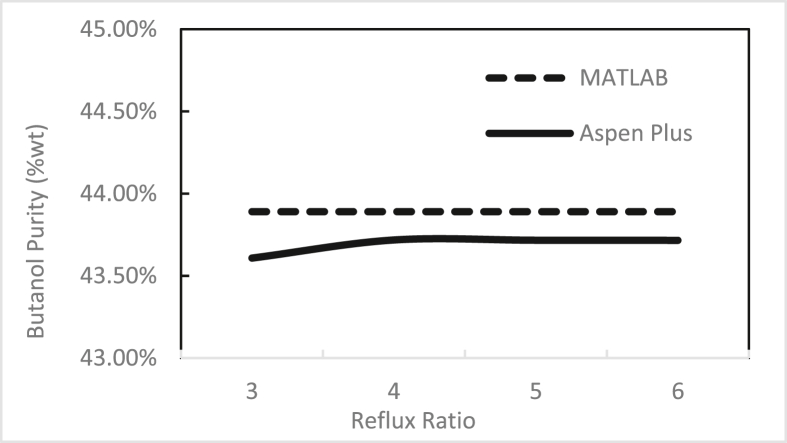
Effect of reflux ratio on the purity of butanol in column 1, feed stage 4, N = 20, D/F = 0.15.

**Figure 5:**
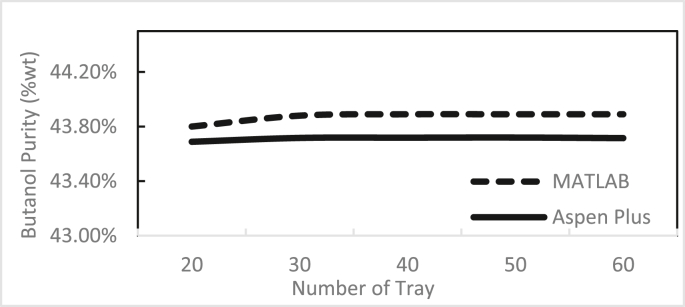
Effect number of trays on the purity of butanol in column 1, R = 4, feed stage 4, D/F = 0.15.

**Figure 6:**
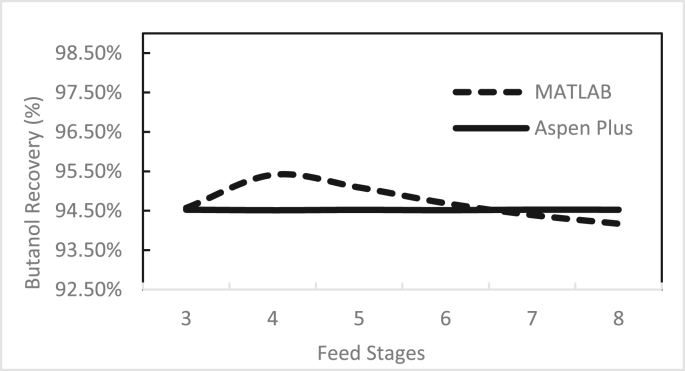
Effect of feed stage on butanol recovery in column 1, R = 4, N = 20, D/F = 0.15.

**Figure 7:**
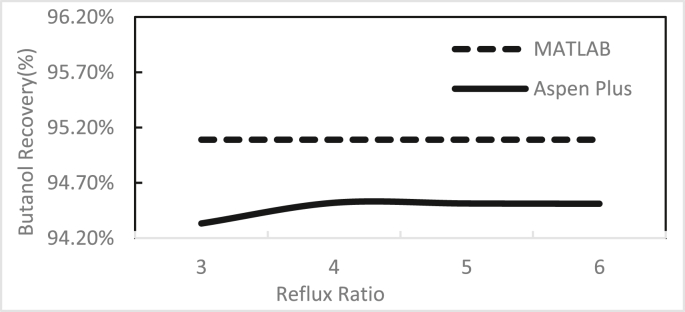
Effect of reflux ratio on butanol recovery in column 1, feed stage 4, N = 20, D/F = 0.15.

**Figure 8:**
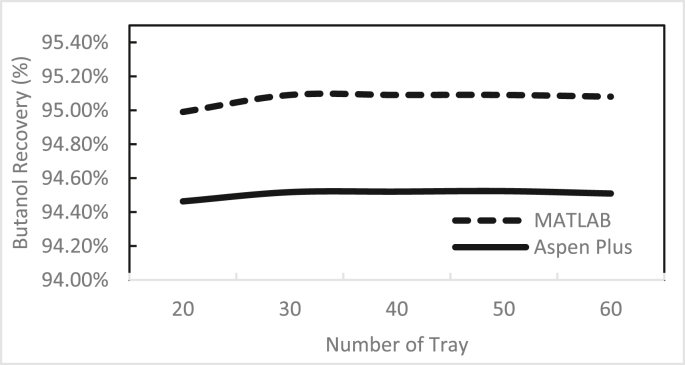
Effect of number of trays on butanol recovery in column 1, R = 4, feed stage 4, D/F = 0.15.

**Figure 9:**
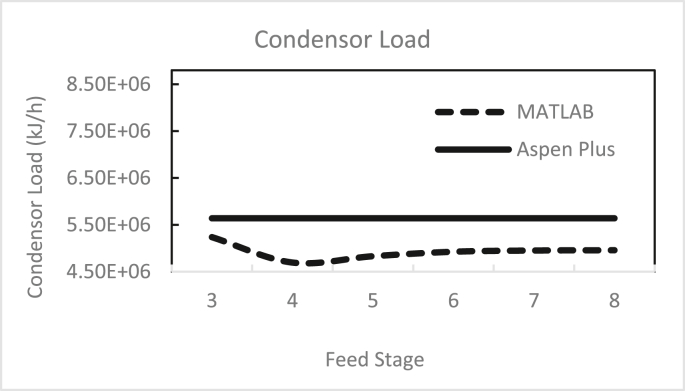
Effect of feed stage on condenser load in column 1, R = 4, N = 20, D/F = 0.15.

**Figure 10:**
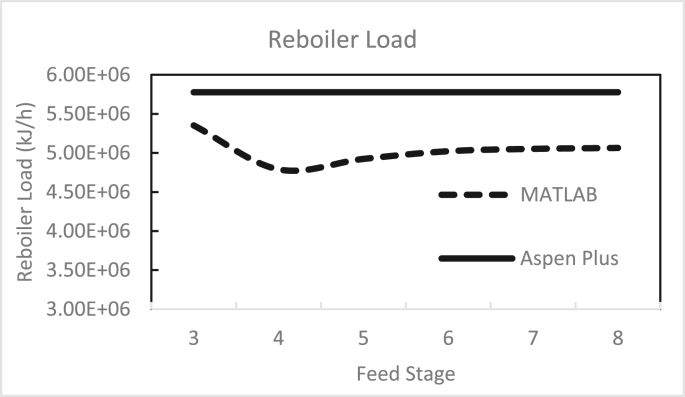
Effect of feed stage on reboiler load in column 1, R = 4, N = 20, D/F = 0.15.

**Figure 11:**
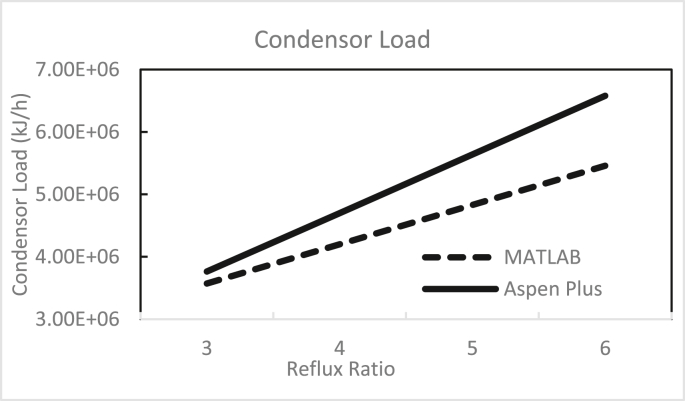
Effect of reflux ratio on condenser load in column 1, feed stage 4, N = 20, D/F = 0.15.

**Figure 12:**
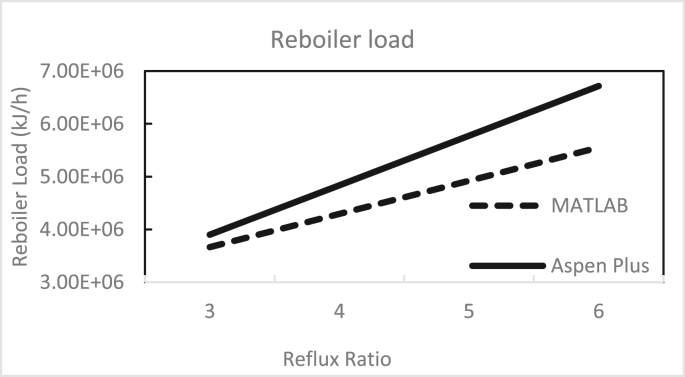
Effect of reflux ratio on reboiler load in column 1, feed stage 4, N = 20, D/F = 0.15.

Figures 3, 4, 5, 6, 7, 8, 9, 10, 11, 12 show the effect of the feed stage, number of stages, and reflux ratio on the butanol purity, butanol recovery, and reboiler and condenser loads in column 1. These figures also present a comparison of the simulation results using the in-house MATLAB-based program versus those obtained with the Aspen Plus simulator. It appears that changing the feed stage location from 3 to 8 does not significantly affect the concentration of butanol in the bottom product, condenser, or the reboiler load when the simulation is performed with Aspen. However, the MATLAB-based program shows the optimum feed location (maximum bottom butanol concentration and minimum reboiler load) at the 4^th^ stage. Using Aspen, increasing the reflux ratio from 3 to 4 slightly increased the butanol concentration, and further increasing the reflux ratio did not significantly affect the butanol concentration. However, using the MATLAB-based program, increasing the reflux ratio from 3 to 6 did not significantly affect the butanol concentration. Increasing the number of trays from 20 to 60 did not significantly affect the bottom butanol concentration, butanol recovery, condenser, or reboiler load. Finally, increasing the reflux ratio from 3 to 6 significantly increased the reboiler load and condenser load. Therefore, it is recommended that column 1 should consist of twenty theoretical stages and should be operated with a reflux ratio of 4, and the feed stage should be the 4^th^ stage. The simulation results using the MATLAB-based program were comparable with those obtained using Aspen Plus.

**Figure 13:**
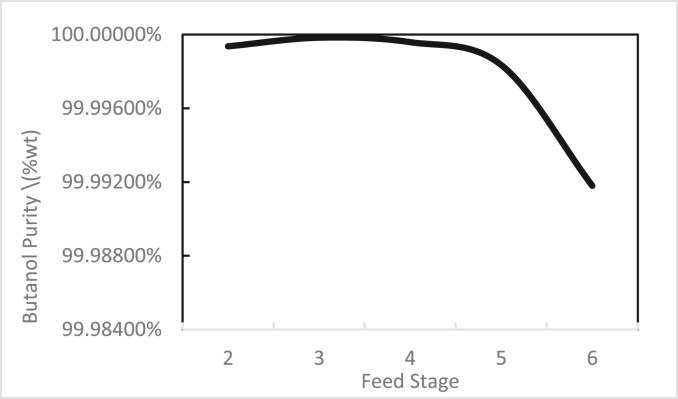
Effect of feed stage on the purity of butanol in column 2, R = 0.4, N = 10, D/F = 0.73.

**Figure 14:**
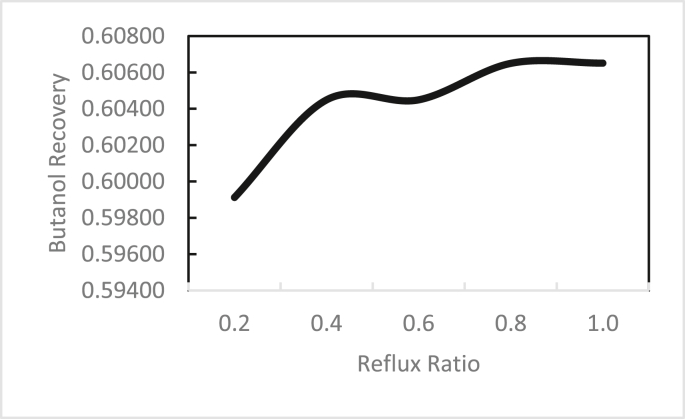
Effect of reflux ratio on butanol purity in column 2, feed stage 2, N = 10, D/F = 0.73.

**Figure 15:**
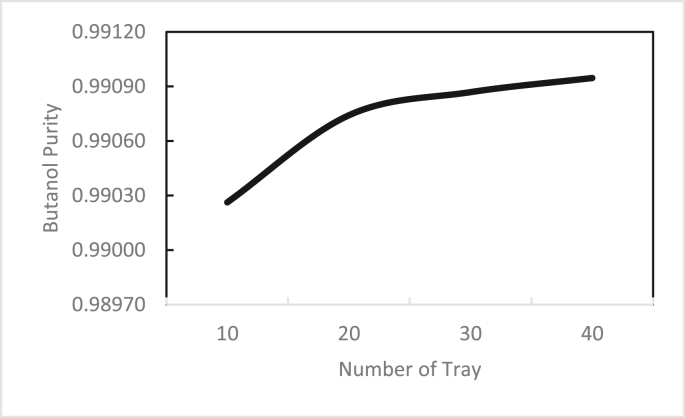
Effect of the number of trays on the purity of butanol in column 2, feed stage 2, R = 0.4, D/F = 0.73.

**Figure 16:**
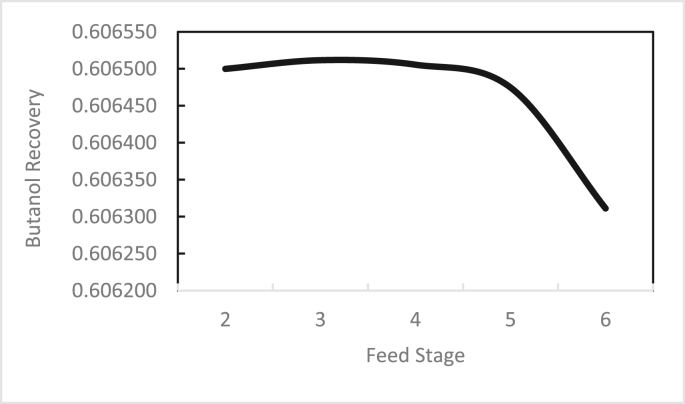
Effect of feed stage on butanol recovery in column 2, R = 0.4, N = 10, D/F = 0.73.

**Figure 17:**
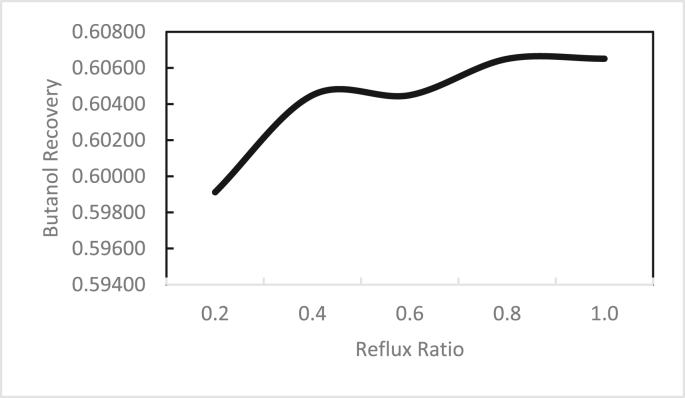
Effect of reflux ratio on butanol recovery in column 2, feed stage 2, N = 10, D/F = 0.73.

**Figure 18:**
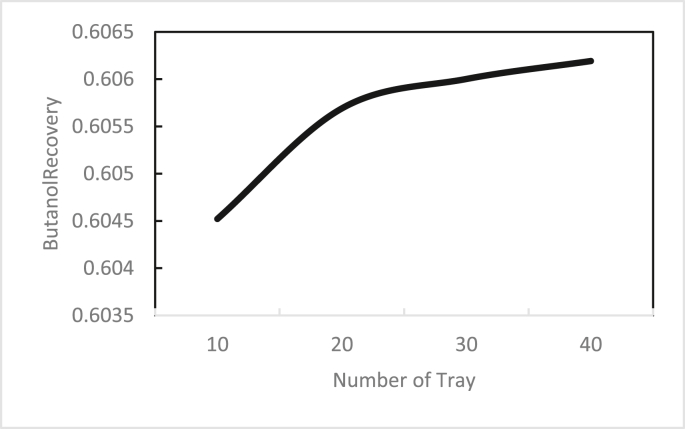
Effect of number of trays on butanol recovery in column 2, feed stage 2, R = 0.4, D/F = 0.73.

**Figure 19:**
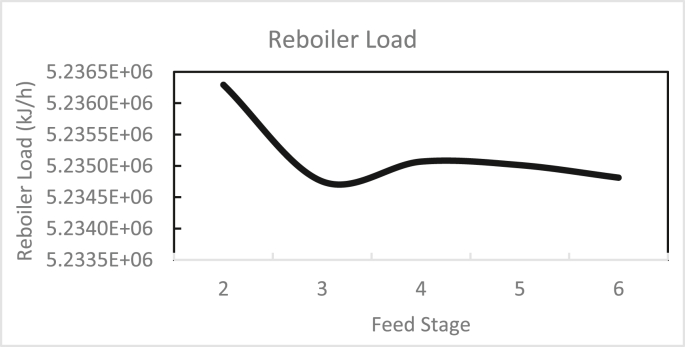
Effect of feed stage on reboiler load in column 2, R = 0.4, N = 10, D/F = 0.73.

**Figure 20:**
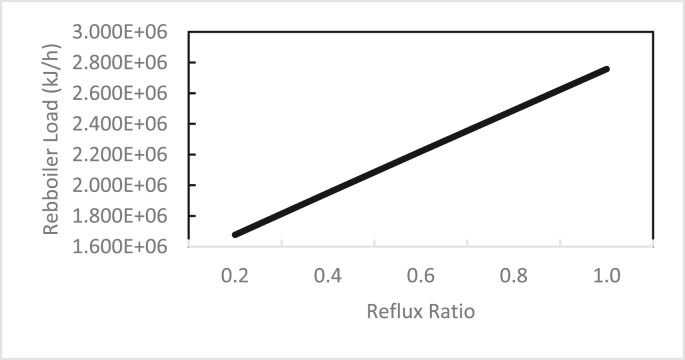
Effect of reflux ratio on reboiler load in column 2, feed stage = 2, N = 10, D/F = 0.73.

**Figure 21:**
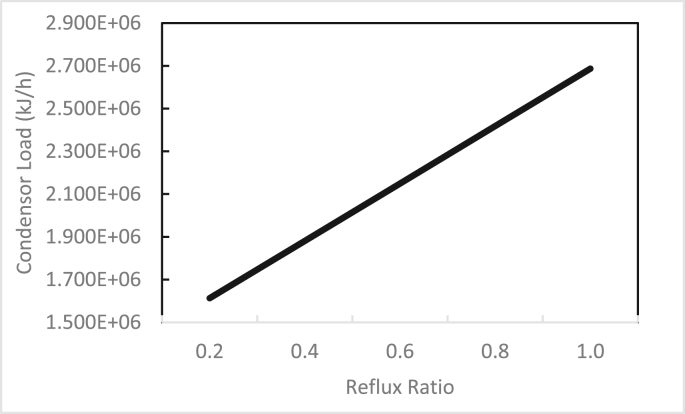
Effect of reflux ratio on condenser load in column 2, feed stage 2, N = 10, D/F = 0.73.

**Figure 22:**
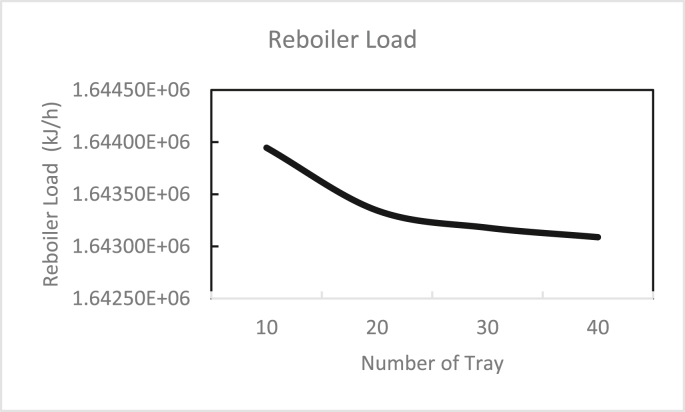
Effect of the number of trays on the reboiler load in column 2, feed stage 2, R = 0.4, D/F = 0.73.

**Figure 23:**
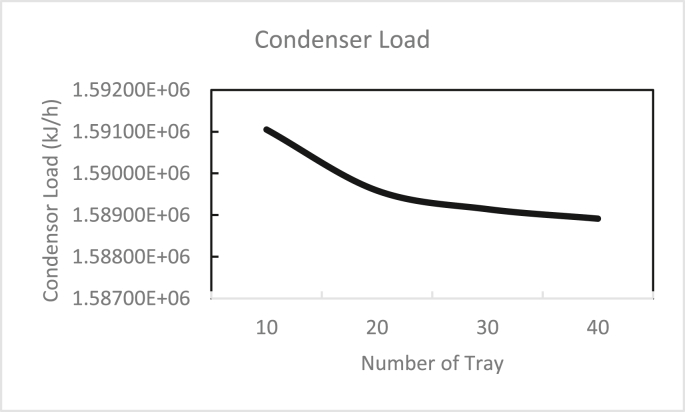
Effect of the number of trays on the condenser load in column 2, feed stage 2, R = 0.4, D/F = 0.73.

In the same way, the effects of the feed stage location, reflux ratio, and number of stages were studied for column 2, as shown in Figures 13, 14, 15, 16, 17, 18, 19, 20, 21, 22, 23. It appears that the purity of butanol approached 100%. To achieve butanol purity greater than 99%, the number of stages required in column 2 must exceed 10. It is recommended that column 2 should be operated with a reflux ratio of 0.4, and the feed stage should be located between the 2^nd^ to 4^th^ stage.

**Figure 24:**
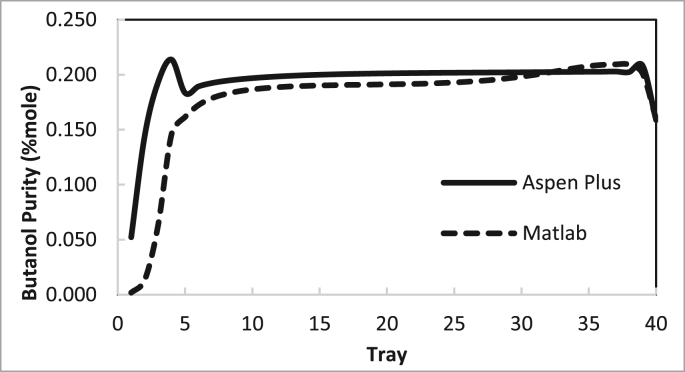
Comparison of the butanol composition profile in column 1, R = 4, feed stage 4, N = 20, D/F = 0.15.

**Figure 25:**
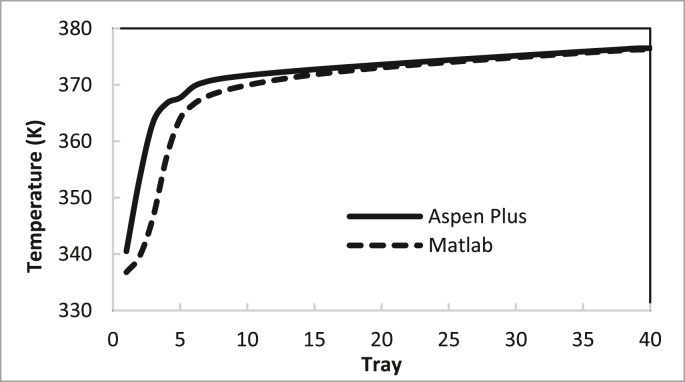
Comparison of temperature profile in column 1, R = 4, feed stage 4, N = 20, D/F = 0.15.

**Figure 26:**
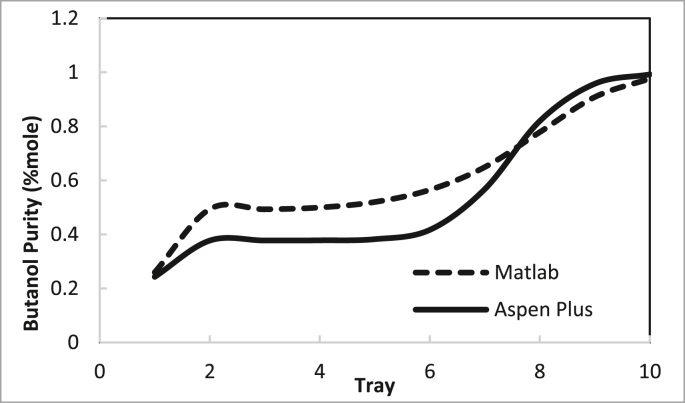
Comparison of butanol composition profile in column 2, R = 0.4, feed stage 2, N = 10, D/F = 0.73.

**Figure 27:**
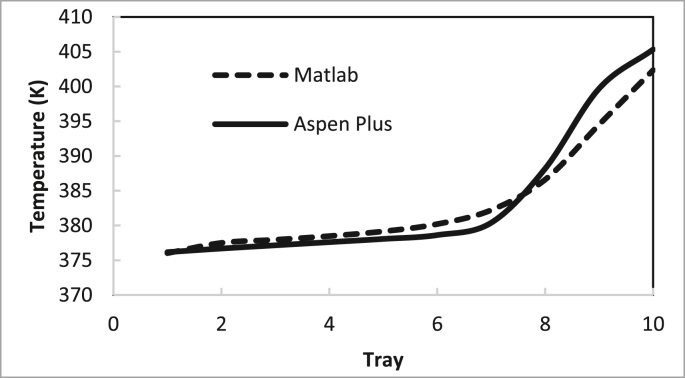
Comparison of temperature profile in column 2, R = 0.4, feed stage 2, N = 10, D/F = 0.73.

From these data, the recommended conditions for the two columns are summarised in Tables 4 and 5. The recommendation was determined by examining the best purity and recovery of butanol and the efficient use of energy. The predicted specific energy consumption in the present study (calculated from the reboiler load for columns 1 and 2 and butanol production rate) was 3.722 MJ (kg butanol)^−1^. Kraemer et al. (2011) estimated the specific energy consumption for hybrid extraction-distillation to separate ABE from fermentation broth as 1.7–3.9 (kg butanol) ^−1^ depending on the type of extraction solvent used. The predicted temperature and concentration profiles in columns 1 and 2 using the in-house MATLAB-based program and ASPEN-PLUS were compared, as shown in Figures 24, 25, 26, 27.

**Table 4:** Recommended Parameter values in Column 1.

Parameter	Value
Feed Pressure (atm)	1.3
Condenser pressure (atm)	1.2
Reboiler pressure (atm)	1.5
D/F Ratio (mole)	0.15
Reflux ratio	4
Number of trays	20
Feed stage	4

**Table 5:** Recommended Parameter values in Column 1.

Parameter	Value
Feed Pressure (atm)	1.5
Condenser pressure (atm)	1.475
Reboiler pressure (atm)	1.7
D/F Ratio (mole)	0.73
Reflux Ratio	0.4
Number of trays	10
Feed stage	2–4

The butanol composition and temperature profiles in each tray of column 1 under the recommended conditions are summarised in Figures 24 and 25, respectively. The average deviation of the butanol composition profile in column 1 was 11%. The average deviation of the temperature profile in column 1 was 0.42%.

The butanol composition and temperature profiles in each tray of column 2 under the recommended conditions are summarised in Figures 26 and 27, respectively.

The average deviation of the butanol composition profile in column 2 is 16%.

The average deviation of the temperature profile in column 2 is 0.43%. The efficiency for column 1 was calculated to determine the actual number of trays using the recommended operating conditions, as shown in Table 3, with O'Connell's correlation. The efficiency of column 1 is 55.43% and the actual number of trays is 70.

The efficiency value of column 2 was calculated to determine the actual number of trays using the recommended operating conditions, as shown in Table 5, with O'Connell's correlation, where the efficiency of column 2 is 54.94% and the actual number of trays is 16. Barrera et al. (2012) studied batch distillation for limonene epoxide recovery, and compared the modelling and simulation data from Matlab and Aspen. The fit between the models was very close, and the Aspen application was easier. However, for the model to be used under all conditions, the necessary assumptions are required. Therefore, mathematical modelling and simulation using MATLAB can help improve the accuracy of the model. The use of Matlab is very flexible. In using the Hang and Wanke method, we modified the convergence criteria and considered the efficiency of the tray.

## 4 Conclusion

The following conclusions can be drawn from this study.

1. A mathematical model of the continuous multicomponent distillation of acetone-butanol-ethanol (ABE) was developed using an equilibrium-based model with the Hang-Wanke method, UNIFAC phase equilibria estimation, and modified convergence criteria using MATLAB R2020a programming language, and simulation was performed using Aspen Plus V9. This study also considered the tray efficiency estimation.
2. The simulation results from Aspen Plus V9 and the mathematical model using MATLAB R2020a showed the same tendency for all variables, along with a similar composition distribution profile, and temperature; thus, this mathematical model is valid for determining the optimum parameters of the acetone-butanol-ethanol (ABE) multicomponent continuous distillation process in a sieve tray column.

## Declarations

### Author contribution statement

L Pudjiastuti, S Nurkhamidah & A Altway: Conceived and designed the experiments; Analyzed and interpreted the data.

T Widjaja: Conceived and designed the experiments; Analyzed and interpreted the data; Contributed reagents, materials, analysis tools or data.

Kornelius Kevin Iskandar, Fikran Sahid & A Pahlevi P: Performed the experiments; Wrote the paper.

### Funding statement

This work was supported by the Deputy for Strengthening Research and Development of the 10.13039/501100009509Ministry of Research and Technology/National Agency for Research and Technology (3/AMD/EI/KP.PTNBH/2020/PKS/ITS/2020).

### Data availability statement

Data included in article/supp. material/referenced in article.

### Declaration of interests statement

The authors declare no conflict of interest.

### Additional information

No additional information is available for this paper.

## References

[bib1] Barrera Rolando, Villa Aída, Correa Consuelo (2012). Modeling and simulation of a batch distillation column for recovering limonene epoxide. Revista EIA.

[bib4] Dadgar A.M., Foutch G.L. (1988). Improving the acetone-butanol fermentation process with liquid-liquid extraction. Biotechnol. Prog..

[bib10] Haigh Kathleen, Petersen Abdul, Gottumukkala Lalitha, Mandegari Mohsen, Naleli Karabo, Görgens Johann (2018). Simulation and comparison of processes for biobutanol production from lignocellulose via ABE fermentation. Biofuel. Bioprod. Biorefin..

[bib12] Jones D.T., Woods D.R. (1986). Acetone-butanol fermentation revisited. Microbiol. Rev..

[bib13] Karimi K., Tabatabaei M., Horvath I.S., Kumar R. (2015). Review paper: recent trends in acetone, butanol, and ethanol (ABE) production. Biofuel Res. J..

[bib14] Kraemer K., Harwardt A., Bronneberg R., Marquardt W. (2011). Separation of butanol from acetone-butanol-ethanol fermentation by a hybrid extraction-distillation process. Comput. Chem. Eng..

[bib17] Liu J., Wu M., Wang M. (2009). Simulation of the process for producing butanol from corn fermentation. Indus. Eng. Res..

[bib18] Lone Sohail (2015). Modeling and simulation of a hybrid process (Pervaporation+Distillation) using MATLAB. J. Chem. Eng. Process Technol..

[bib20] Luyben W.L. (2008). Control of the heterogeneous azeotropic n-butanol/water distillation system. Energy Fuels.

[bib21] Marlatt J.A., Datta R. (1986). Acetone-butanol fermentation process development and economic evaluation. Biotechnol. Prog..

[bib26] Park C.H., Geng Q.H. (1992). Simultaneous fermentation and separation in the ethanol and abe fermentation. Separ. Purif. Methods.

[bib27] Patil Dr, Kiran, Kulkarni Bhaskar. (2014). Mathematical modeling and simulation of reactive distillation column using MATLAB and aspen Plus®. Int. J. Lat. Trend. Eng. Sci. Technol..

[bib31] Qureshi N., Blaschek H.P. (2000). Economics of butanol fermentation using hyper-butanol producing clostridium beijerinckii ba 101. Trans. Inst. Chem. Eng., Part C.

[bib32] Qureshi N., Blaschek H.P. (2001). Recovery of butanol from fermentation broth by gas stripping. Renew. Energy.

[bib33] Qureshi N., Blaschek H.P. (2001). ABE production from corn: a recent economic evaluation. J. Ind. Microbiol. Biotechnol..

[bib35] Sari N.K. (2011). Simulation and experimental system terner acetone-butanol-ethanol with batch distillation. Art. Bali Int. Sem. Sci..

[bib36] Sari N.K. (2011). Validasi simulasi sistem terner aseton-butanol, ethanol (ABE) dengan sistem terner methanol-ethanol-propanol (MEP). Prosiding Seminar Nasional Fundamental dan Aplikasi Teknik Kimia.

[bib37] Sari N.K., Wibawa G., Kuswandi, Soewarno N., Renanto (2007). Pemisahan sistem biner ethanol-air dan sistem terner aseton-n-butanol-ethanol-dengan distilasi batch sederhana. Jurnal Ilmiah Sains dan Teknologi.

[bib38] Seader J.D., dan Henley E.J. (2006). Separation Process Principles.

[bib39] Sinnot R.K. (2005). Coulson & richardson’s chemical engineering.

